# Early‐life influences on the risk for later‐life Alzheimer's and non‐Alzheimer's dementia: A nearly full life course prospective cohort study

**DOI:** 10.1002/alz.70967

**Published:** 2026-01-15

**Authors:** Pamela Herd, Kamil Sicinski, Victoria Williams, Sanjay Asthana, Michal Engelman

**Affiliations:** ^1^ Ford School of Public Policy University of Michigan‐Ann Arbor, Joan and Sanford Weill Hall Ann Arbor Michigan USA; ^2^ Center for Demography of Health and Aging University of Wisconsin‐Madison Madison Wisconsin USA; ^3^ Department of Medicine University of Wisconsin School of Medicine and Public Health Madison Wisconsin USA; ^4^ Department of Medicine University of Wisconsin School of Medicine and Public Health Madison Wisconsin USA; ^5^ Department of Sociology University of Wisconsin‐Madison Madison Wisconsin USA

**Keywords:** cognitive reserve, early life, educational attainment, social determinants, social exposome

## Abstract

**BACKGROUND:**

Dementia prevention research has largely used educational attainment as a proxy for early‐life. Given the known influence of early‐life exposures on brain development, more attention to early‐life exposures is warranted.

**METHODS:**

We employ the Wisconsin Longitudinal Study, a nearly full life course cohort study, to examine the influence of prospectively measured early‐life risk factors for dementia in later life.

**RESULTS:**

We find that early‐life risk factors that precede high school completion, rather than early adulthood post‐secondary schooling, exert influence on later‐life dementia. Household parental resources influence non‐Alzheimer's disease (AD), but not AD, dementia risk. In contrast, markers for adolescent cognitive reserve (cognitive and academic performance measures) influence AD dementia risk, in part, by modifying genetic risk.

**DISCUSSION:**

Using education as a proxy for early‐life exposures conceals specific mechanisms that influence distinct dementia etiologies and are separately intervenable. Education's influence may be confined to the early‐life and adolescent period, where brain development is especially malleable.

**Highlights:**

- Educational attainment is commonly used as a proxy for early‐life risk factors for dementia.
- Given known early‐life influences on brain development, more attention to the period is warranted.
- Early‐life parental income influences non‐AD dementia risk.
- Early‐life cognition and academic performance influence AD dementia risk.
- Using educational attainment as a proxy for early‐life exposures conceals separate intervenable risk factors.

## 1 BACKGROUND

The 2024 Lancet Commission on dementia summarized modifiable risk factors for dementia across the lifespan.^1^ While early‐life exposures are known to influence brain development, the commission highlighted just one early modifiable risk factor, educational attainment, stating that “a long duration of education is beneficial.”^1^ In contrast, the report highlighted robust research on midlife modifiable behaviors, ranging from behavioral risks (like sedentary lifestyle, alcohol consumption, and social isolation) to emergent health risks (like hypertension, diabetes, cardiovascular diseases, and strokes).^1^

**FIGURE 1:**
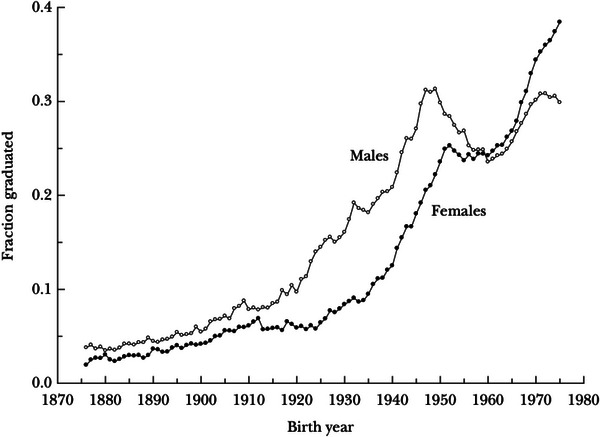
College graduation rates for men and women: cohorts born from 1976 to 1975.*Source*: Goldin C, Katz LF, Kuziemko I. The homecoming of American college women: The reversal of the college gender gap. Journal of Economic Perspectives. 2006 Sep 1;20(4):133‐56.

The Lancet report accurately represented existing evidence on early life, where dementia studies typically only examine educational attainment.^1^ But because post‐secondary schooling extends into people's 30s, attainment captures multiple periods, including early life, adolescence, and into adulthood.^2^ While older cohorts had very limited post‐secondary schooling, this is changing rapidly.^2^ As Figure 1 shows,^3^ cohorts now reaching ages characterized by accelerated dementia risk have substantial post‐secondary schooling. Educational attainment captures events in both childhood and early adulthood.^4^, ^5^, ^6^, ^7^

More attention to early life, from childhood through adolescence, is warranted, given overwhelming evidence that it's a “critical period” for brain development.^8^ The period is fundamental to establishing cognitive reserve – and the prevention of dementia, even in the face of underlying pathology.^9^

There's accumulating evidence that early‐life contextual and individual resources shape cognitive function over the life course.^10^, ^11^, ^12^ Contextual resources may influence whether children develop cognitive reserve. Children raised with fewer socioeconomic resources have a great risk for lower levels of cognitive ability by late adolescence, including measured differences in brain structure and functioning.^13^, ^14^, ^15^, ^16^ This in turn affects their academic performance, gaining fewer cognitive benefits from each year of schooling.^17^ Indeed, a few studies show early‐life cognitive and academic performance, which may serve as a proxy for cognitive reserve, do predict mid‐ to later‐life cognitive function.^5^, ^6^, ^7^, ^18^

While there is substantial interest in understanding early‐life influences on dementia, existing studies are hampered by three challenges. First, with few exceptions, studies lack prospectively collected data that capture high‐quality early‐life measures and later‐life dementia measures; studies might have one or the other, but rarely both.^19^, ^20^, ^21^, ^22^ Retrospective childhood measures, especially economic, tend to be unreliable, possibly underestimating their influence.^23^

A second limitation is the reliance on educational attainment as a proxy for childhood factors.^1^ Attainment may conceal specific early‐life mechanisms, like parental socioeconomic resources or markers of early‐life cognitive reserve, such as cognitive function (e.g., IQ) or secondary school academic performance, which are separately intervenable. Using educational attainment also muddies “when” to intervene. Is education's influence confined to the childhood and adolescent period, where brain development is especially malleable? Or does subsequent post‐secondary schooling, which can span well into adulthood, further enhance the protective effect? Most studies have too few participants who have substantial post‐secondary schooling, given that it is only recent cohorts that have, in large numbers, achieved post‐secondary schooling.

Finally, studies focused on early life, including educational attainment, rarely capture etiological differences in dementia. But the influence of early‐life factors may vary depending on whether the outcome is a vascular or non‐vascular dementia. For example, there's evidence that the environment has a stronger influence on vascular dementias, while genetics has a stronger influence on non‐vascular dementias.^24^, ^25^ Early‐life covariates that mostly capture “environment,” such as childhood poverty, versus those that have known partial genetic influences, such as early‐life cognitive resources, may differentially correlate with AD versus non‐AD dementias.^26^, ^27^, ^28^

Consequently, we draw on a life‐course longitudinal study, with prospectively collected early‐life measures and robust later‐life dementia measures, to test three hypotheses. First, we posit that early‐life parental resources and markers for cognitive reserve (adolescent cognitive ability and academic performance) will negatively correlate with increased risk for late‐life dementia. Second, we hypothesize that post‐secondary schooling, which captures early adulthood rather than early life, will not independently correlate with dementia risk. Third, we posit (a) that early‐life contextual factors, specifically parental resources, will have a stronger association with non‐AD dementias than AD dementia and (b) that early‐life cognitive academic performance, which proxies for cognitive reserve, will modify genetic risk for AD, but not non‐AD dementias.

## 2 METHODS

### 2.1 Data

The Wisconsin Longitudinal Study (WLS) provides a unique opportunity to explore the relationship between early‐life, post‐secondary schooling and dementia in later life. It has three key advantages. First, it is the only nearly full life‐course longitudinal US‐based sample of older adults that includes prospectively collected measures of adolescent cognitive functioning, as well as other key prospectively collected measures of health and cognition. Second, it is a cohort design focused on those born around 1939, which helps rule out, for example, period effects that might confound analyses focused on educational disparities. The WLS is uniquely situated to explore the questions posed in this study.^29^

**RESEARCH IN CONTEXT**

1. **Systematic review**: The authors used PubMed to review existing research on early‐life risk factors for AD and non‐AD onset. The review revealed that dementia prevention research has paid limited attention to the early‐life course, despite its being a period marked by significant brain plasticity. Most research relies on a single variable, educational attainment, as a proxy for early life.
2. **Interpretation**: Our findings indicated that using education as a proxy for early‐life exposures conceals specific mechanisms that influence distinct dementia etiologies and are separately intervenable. Moreover, we find that education's influence may be confined to early‐life and adolescent periods, when brain development is especially malleable.
3. **Future directions**: The manuscript emphasizes the need to better understand how early life alters the risk for dementia in later life. This requires the collection of full life‐course data that capture both prospective measures of early‐life factors and detailed measures of dementia etiologies in later life.

The WLS is based on a randomly selected one‐third sample of all 1957 Wisconsin high school graduates and a randomly selected sibling for each eligible graduate.^30^ Participants were originally empaneled at age 18 (1957), which was followed by data collection at ages 25 (1964), 36 (1975), 54 (1993), 65 (2003 to 2004), and 72 (2011 to 2012). Data on cognition, which are detailed below, are drawn from administrative records (standardized IQ tests administered to all Wisconsin high school students starting in the 1920s) and from cognitive assessments administered in 2011.

Measures of adolescent cognition and our dementia outcomes were available for 4514 sample members who completed the 2021 data collection. These included both the original graduate respondents as well as siblings of those graduates. The original sample frame included a random 80% subsample of WLS respondents who had participated in either the 2005 or 2011 rounds of data collection. The random subsample had received a greater array of cognitive assessments in 2005 and 2011. Because they were randomly selected, this should not lead to any selection effects driving differences across these outcomes. The 2021 WLS‐ILIAD response rate was 89%, with 160 participants missing due to attrition and 433 due to mortality.

#### 2.1.1 Outcome measures

Dementia status: In 2021, dementia prevalence was determined using a previously described multiphase approach.^31^ Briefly, all eligible WLS participants were administered a validated telephone‐based cognitive screening measure (TICS‐m) to screen for individuals at risk for cognitive impairment and dementia. Those scoring below an established cutoff of 29 were selected to undergo more detailed neuropsychological and comprehensive medical evaluation, with each case reviewed individually during a multidisciplinary consensus conference (consisting of a neuropsychologist, geriatrician, and nurse practitioner) to determine dementia status and likely underlying etiology. For participants who were deceased or unable to complete the assessment, dementia status was algorithmically defined using information collected by proxy interview using the Dementia Questionnaire and validated by clinician review.^31^

#### 2.1.2 Early‐life socioeconomic resources

Early socioeconomic resources: (1) Family income was imputed using occupational and earnings information from the 1940 census and WLS, including tax records. We then applied an inverse hyperbolic sine transformation with the scaling parameter estimated by maximum likelihood to maximize fit in a parental earnings regression; (2) mothers’ educational attainment was measured in 1964 and 1975 based on the highest degree completed. Sensitivity analyses, as well as prior empirical research, indicate mother's education was the best marker for parental educational attainment (analysis available on request). Paternal education was also measured in 1964 and 1975 based on the highest degree completed.

#### 2.1.3 Early‐life cognition and academic performance

Adolescent measure of cognitive functioning: WLS is one of just a few longitudinal aging cohort studies with an early‐life cognitive measure. This measure is derived from the Henmon–Nelson IQ test administered to WLS participants during their junior year in high school (1956). It was a 30‐min test consisting of 90 items in order of increasing difficulty. It included vocabulary, sentence completion, disarranged sentences, classification, logical selection, series completion, directions, analogies, anagrams, proverb interpretation, and arithmetic problems. It highly correlates (0.83) with IQ tests more commonly administered today, especially the WAIS.^32^

Adolescent academic performance: This measure is students’ high school rank, which is a percentile rank based on high school grades (100−[(Rank in class / [No. of students in class]) × 100]). This was collected prospectively from high school administrative data in 1957. It reflects participants’ cumulative academic performance in high school. While the high school grade percentile rank is not available for siblings, both graduates and siblings were asked to rate their grade point average as falling in the top quarter, bottom quarter, or somewhere in the middle in the 2011 round of interviews. We leveraged these data to impute the sibling high school rank in a separate model using adolescent cognition, education, parental resources, and sex as covariates. The analysis variable is standardized to a mean of zero and a standard deviation of 1.

#### 2.1.4 Covariates and moderators

We controlled for age and sex. Note that there is very limited variance in age for the graduate sample, where all graduated from high school in 1957. Approximately 90% of siblings fall on either side of the graduate's age by 7 years. In addition, we included the following measures:

Genetic risk: Rather than controlling for APOE ε4 status, we adopted a non‐linear, continuous risk score developed by Vasiljevic and colleagues.^33^ The score is based on a sample of non‐Hispanic Caucasian participants from the Wisconsin Registry for Alzheimer's Prevention study and is derived from odds ratios of the six combinations of the ε2/ε3/ε4 genotype. We do want to emphasize that APOE ε4 status was not used to establish the research diagnosis for dementia in this study.

Educational attainment: The first detailed educational attainment report in WLS came from parents in 1964. In 1975, respondents themselves reported on all schooling obtained since graduating high school. In every subsequent wave, participants were asked retrospective questions regarding any changes in their educational attainment (including additional years of schooling, even if no additional degree was obtained). Over half of the sample did not pursue education past high school. We employed a summary measure that reflected the highest degree of completed post‐secondary schooling.

### 2.2 Statistical models

We employed multinomial logit models to analyze the relationship between childhood and adolescent risk factors and later‐life dementia risk. The dependent variable for the primary models categorizes participants into one of three diagnostic groups based on the study's cognitive assessments: Alzheimer's disease (AD) dementia, non‐AD dementia, and individuals classified as no dementia or impairment. Sensitivity analyses included models where the reference category only included individuals who scored above the cut‐off for dementia on the TICS‐M and, hence, indicated no sign of cognitive impairment. The findings remained consistent, with only small variation, such as the size of standard errors for some findings. Table A1 presents the full model estimates, including those for individuals who scored below the cut‐off for dementia on the TICS‐M, but were then assessed as having mild cognitive impairment.

Non‐response to the Iliad survey related to pre‐existing cognitive problems or poor health, which could have biased our results. To address this concern, we constructed a post‐stratification weight with respondents to the 2011 survey round serving as the reference population. The response propensities were estimated using a logistic regression with a rich set of covariates covering health, cognition, socioeconomic status, and family background. Missing observations were imputed using IVEware, and trimming was applied at the 5th and 95th percentiles to guard against bias from extreme weights.^34^

## 3 RESULTS

### 3.1 Study participant demographics

Table 1 provides descriptive statistics about the sample. Around 10% of the sample was classified as meeting criteria for dementia, which included 7% (*n* = 374) clinically attributed to suspected AD and 2% (*n* = 100) thought to be primarily due to non‐AD pathology. Participants’ median age was 81 when dementia was assessed in 2021. The average educational attainment for WLS participants is 14 years; that is, the median participant has 2 years of post‐secondary schooling. Reflecting the age of the sample, 55% of participants are women. Parental median income when participants were in high school was $5600 a year, which is $600 more than the US median family income in 1957.^35^

**TABLE 1:** Descriptive statistics (*N* = 4514).

	Mean/Proportion	SD	Minimum	Maximum	IQR	Median
*Cognition summary outco me*						
No dementia	0.91					
Non‐Alzheimer's dementia	0.02					
Alzheimer's dementia	0.07					
APOE score	0.29	0.63	−0.70	2.56	0.77	0.00
Indicator for APOE score imputation	0.11					
*Parental resources*						
Mother's education	10.63	2.73	5.00	16.00	4.00	12.00
Parental income, 1957 (1000s)	6.59	6.34	.30	99.80	4.00	5.60
Female	0.55	0.50	0.00	1.00	1.00	1.00
Age at time of screening (2021)*	80.28	3.50	59.00	98.00	1.00	81.00
Years of education	13.99	2.46	8.00	20.00	4.00	12.00
Adolescent cognition	103.95	14.70	61.00	145.00	19.00	104.00
High school rank	103.62	14.70	50.96	150.94	20.00	104.00

Abbreviations: APOE, apolipoprotein E; IQR, interquartile range; SD, standard deviation.

* Participants were screened in 2021.

### 3.2 Early‐life correlates of later‐life AD and non‐AD onset

Table 2 provides the results from multinomial logit models. The coefficients are presented as odds ratios. Overall, the results demonstrate that childhood and adolescent family resources and achievement are robust predictors of dementia in later life. Post‐secondary schooling, however, is not independently associated with dementia.

**TABLE 2:** Dementia regressed on childhood parental resources, adolescent achievement, and young adulthood educational attainment (*N* = 4514).

	Non‐AD dementia			AD dementia		
Childhood parental resources						
Mother's education	0.92 [0.84,1.01]	0.92 [0.84,1.01]	0.92 [0.84,1.01]	1.03 [0.97,1.10]	1.06 [0.99,1.13]	1.05 [0.99,1.12]
Parental income	0.78 [0.63,0.98]	0.78 [0.62,0.98]	0.79 [0.63,1.00]	0.90 [0.80,1.00]	0.94 [0.84,1.06]	0.93 [0.82,1.04]
Adolescent cognition		1.09 [0.82,1.45]	1.14 [0.82,1.59]		0.65 [0.55,0.77]	0.76 [0.62,0.93]
High school rank			1.07 [0.76,1.50]			0.71 [0.58,0.86]
Years of post‐secondary schooling			0.81 [0.61,1.09]			1.17 [0.98,1.39]
Demographics and genetic risk						
Female	0.58 [0.33,0.99]	0.58 [0.34,1.00]	0.53 [0.29,0.99]	1.18 [0.88,1.57]	1.19 [0.88,1.59]	1.46 [1.07,2.00]
Age in 2021^*^	1.20 [1.10,1.30]	1.19 [1.10,1.28]	1.18 [1.10,1.28]	1.22 [1.16,1.28]	1.22 [1.16,1.28]	1.22 [1.16,1.28]
Genetic Risk Score	1.29 [0.88,1.91]	1.30 [0.88,1.90]	1.29 [0.87,1.89]	2.09 [1.70,2.56]	2.08 [1.68,2.57]	2.11 [1.71,2.60]

*Note*: Multinomial logit model with odds ratios. 95% confidence intervals in brackets. The reference category is those without dementia.

* Assessments conducted in 2021.

The left side of Table 2 focuses on the risk of diagnosis for non‐AD dementias. Children in households with greater parental resources, including mothers having higher educational attainment and parents having greater income, are less likely to have non‐AD dementias. Each additional year of a mother's schooling reduces the odds by 8%. Sensitivity analyses showed that the father's educational attainment was not a significant predictor of dementia etiology.

The findings, however, are more robust for the prospectively collected measure of parental income than for mothers’ educational attainment, given lower standard errors and larger effect sizes. Here, each one‐unit change in log income reduces the odds by just over 20%. There is no relationship, however, between adolescent achievement, high school academic performance and adolescent cognition, or post‐secondary schooling, and subsequent risk for non‐AD dementia. Models that included cognition and academic performance separately did not alter these findings.

The right side of Table 2 focuses on AD dementia as an outcome. Here, we see robust associations between adolescent achievement and AD dementia, but weak or no associations between parental resources and AD dementia. For each standard deviation increase in high school rank, there is a corresponding 29% reduction in the odds of AD dementia. The association with adolescent cognition is slightly weaker, with somewhat larger standard errors. For each standard deviation increase in adolescent cognition, which was measured prospectively, there is a 24% reduction in the odds of an AD dementia diagnosis.

There were no meaningful associations, however, between post‐secondary schooling and AD dementia diagnoses. Before the inclusion of adolescent achievement measures, there was a weak association between parental income and AD dementia diagnoses, with those having higher income having a lower odds of AD dementia.

**FIGURE 2:**
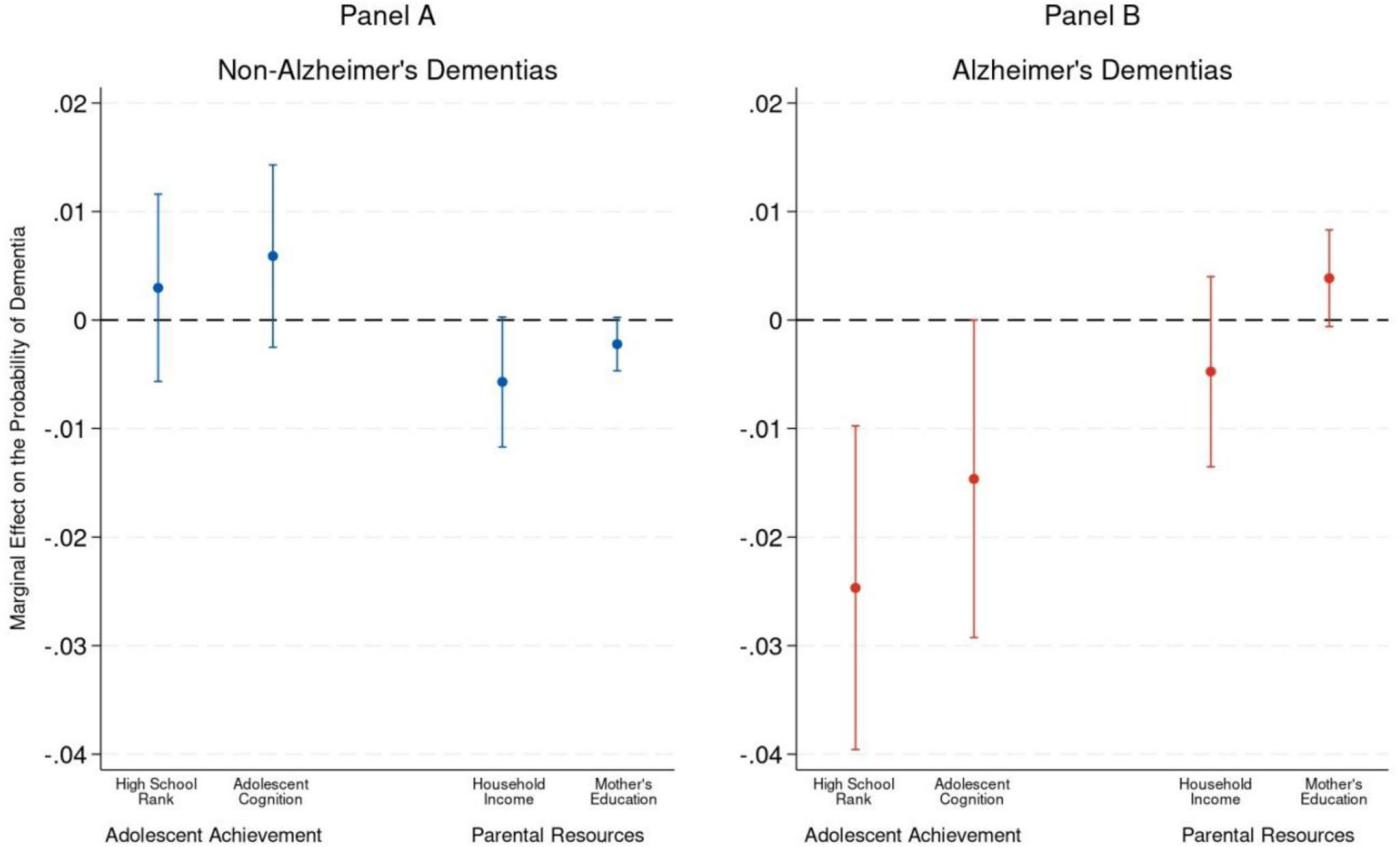
Early‐life predictors of non‐Alzheimer's and Alzheimer's dementia onset.

Figure 2 provides estimates that reflect the probability of AD and non‐AD risk, rather than the odds as presented in Table 2. For each 10‐percentage‐point increase in high school rank, the probability of AD dementia declined by 2.5 percentage points. The models control for all covariates, including demographic, genetic, academic adolescent achievement, and early‐life parental resources.

### 3.3 Early‐life moderators of genetic risk for later‐life AD dementia

**FIGURE 3:**
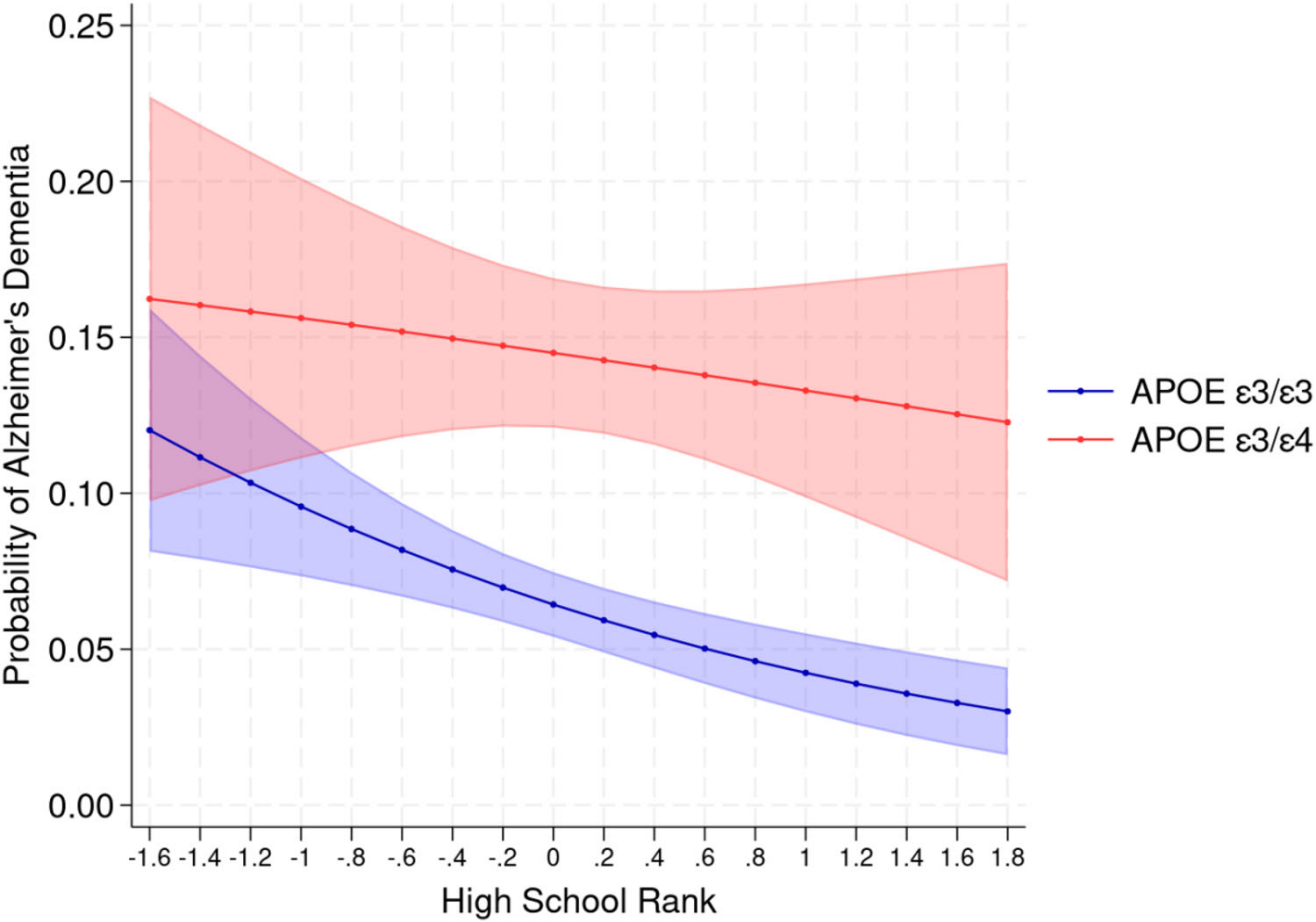
Interaction between AD genetic risk and high school academic performance.

We also tested interactions between early‐life academic achievement and genetic risk on dementia outcomes. For non‐AD dementias, given that neither main effects for genetic risk nor academic achievement were statistically significant, it is not surprising that there is no evidence of an interaction. As Figure 3 shows, however, including adjustments for all covariates, having a higher adolescent academic performance modifies the influence of genetic risk on risk for AD dementia, especially softening the risk for AD dementia among those without the *APOE* ε4 allele. The interaction between genetic risk and adolescent cognition was not statistically significant, though showed a similar trend.

## 4 DISCUSSION

Our study aimed to identify early‐life risk factors for dementia in later life. We used a unique, nearly full life‐course longitudinal dataset whose participants are currently in their 80s and have been assessed using a rigorous protocol for determining dementia.

We found that parental resources and markers for adolescent cognitive reserve were robustly associated with late‐life dementia. More specifically, we found that parental resources, especially a prospectively collected measure of parental income, influenced non‐AD dementia risk, while adolescent cognition and academic performance influenced AD dementia risk. We found no evidence, however, that post‐secondary schooling was protective against any form of dementia. Capturing differential dementia etiologies is important for parsing out these relationships. The failure to distinguish etiologies may overestimate the influence of adolescent cognition and academic performance and underestimate the influence of parental resources like income, which operate earlier in the life course and may influence both adolescent cognition and academic performance.

Examining the influence of prospectively collected measures that capture cognitive and academic performance in adolescence provided a novel opportunity to understand how early‐life factors may influence dementia risk in later life, as well as how to intervene in early life to prevent dementia. Measures of adolescent academic performance and cognitive function capture the culmination of a critical period for brain and cognitive development in early life – and a few studies have shown they influence cognitive function in mid and later life.^5^, ^6^, ^7^, ^13^

Existing studies, however, did not attend to how early‐life cognition and academic performance influenced the risk for dementia. A small number of studies captured early‐life cognition, and they did find an influence on dementia, but they did not also capture people's schooling experiences or a more direct measure capturing students' cumulative crystallized knowledge and academic skills.^15^, ^36^, ^37^, ^38^, ^39^, ^40^, ^41^ We found that both of these adolescent markers, one a more typical measure of cognitive ability, the other capturing the kind of crystalized knowledge and skills one gains in school, had large independent influences on the risk for AD dementia in late life. Importantly, both of these early‐life factors are intervenable.

There is robust evidence that we can intervene in ways that enhance childhood cognitive development and academic skills. Cognitive development in childhood is heavily influenced by the environment, ranging from the quality of early education environments to the quality of their home environments.^42^, ^43^ An early life characterized by safe and nurturing environments, adequate nutrition and shelter, and high‐quality education enhances cognitive development throughout childhood, as well as how students actually perform academically.^44^, ^45^

Academic performance in school also appears protective against an AD dementia diagnosis and is also separately intervenable. While cognitive ability tests and high school academic performance are correlated, they are not interchangeable.^46^,^47^ School quality and related supports all have important influences on what children learn in school, as measured by their overall academic performance.

Our findings regarding the limited protective influence of post‐secondary schooling must be understood in the context of the existing research on these relationships. In short, we do find that schooling matters, in particular, adolescent academic performance clearly impacts AD dementia risk, but it is the timing of that schooling that likely matters more. Indeed, mandatory schooling laws, which kept students in school until age 16, are associated with improved cognitive function in mid to early late life.^48^, ^49^ Most of the existing empirical work, however, is focused on variation in schooling prior to the completion of high school, largely because so few individuals completed post‐secondary schooling in older cohorts.^1^ It is only recent cohorts, now reaching the ages where they are experiencing rapid risk for dementia, that have significant amounts of post‐secondary schooling.^23^

Notably, our findings emphasize the importance of how dementia etiologies are assessed. For example, similar to this study, Manly and colleagues (2022) found no protective effect of college attainment compared to those with high school degrees, whereas other investigators, also using the Health Retirement Study, did find evidence of a protective effect of a college degree.^50^, ^51^, ^52^, ^53^ The key difference between these studies was that Manly and colleagues captured functional impairment, as well as cognitive performance, in their assessments for dementia, whereas the other studies relied exclusively on a dementia short item screener (TICS‐m), which only captures cognitive performance. These differences across measurements may provide important clues as to these underlying relationships.

Our findings also emphasize the importance of attending to differing dementia etiologies. We found important differences in how early‐life factors influenced non‐AD versus AD dementias. Non‐AD dementias were not predicted by individual‐level academic achievement, including measures of both adolescent cognition and academic performance. Prior research is mixed on the relationship between early‐life cognition and later‐life health risks that precipitate vascular dementias, like strokes and cardiovascular disease.^54^, ^55^, ^56^ Indeed, among the few existing studies examining these relationships, there is no association between early‐life IQ and stroke risk.^57^ IQ has been shown to be predictive of cardiovascular disease, but concentrated only among those with low IQ, and studies have found that accounting for early‐life socioeconomic resources and educational attainment renders associations non‐significant.^58^, ^59^, ^60^, ^61^ This may explain why neither adolescent cognitive function nor academic performance correlates with non‐AD dementia etiologies.

In contrast, we found evidence that parental socioeconomic resources, especially parental income, may promote resistance to non‐AD dementia. Unlike nearly all prior studies, we have a prospectively collected measure of parental income. Both parental income and mother's educational attainment were predictive of non‐AD dementia. Unlike with early‐life cognitive function, there is robust evidence that both early‐life economic conditions and parental educational attainment are predictive of strokes and cardiovascular disease in later life. Consequently, these early‐life socioeconomic resources may reduce long‐term risk for non‐AD dementias by reducing the risk for both strokes and cardiovascular disease. In short, these early‐life resources may actually promote resistance to non‐AD dementias by preventing subsequent vascular damage caused by strokes and cardiovascular disease.^62^, ^63^, ^64^

For AD etiologies, in contrast to non‐AD dementias, we found that both adolescent cognition and academic performance predicted AD dementia risk, while household parental resources did not. Importantly, we found that higher adolescent academic performance, compared to lower adolescent academic performance, modified the risk for developing AD dementia in later life. We know of no other study that tested these interactions. While we cannot confirm that this reflects resistance to the formation of these pathologies, as opposed to resilience to underlying AD pathologies, we are currently collecting venous blood samples that will be assayed for AD biomarkers, thus allowing us to more directly test whether these findings reflect resistance or resilience. Moreover, it's important to emphasize that some individuals diagnosed wtih AD dementia also have evidence of vascular dementia.

There are some important limitations to this study. WLS is a sample of mostly White high school graduates. That said, over 80% of Wisconsin residents in the corresponding birth cohort did complete a high school degree, and the educational and racial composition of the WLS sample is thus broadly representative of its generation. While they are a slightly more advantaged group relative to the general population, it is important to note that just over 20% of the WLS sample grew up in a poor household.^65^ Moreover, because our sample excluded some disadvantaged individuals, our findings likely underestimated the influence of early‐life conditions on dementia risk. That said, these findings may not hold for other demographic or geographic groups. Notably, however, the focus on high school graduates is applicable to more recent cohorts and allows for sufficient statistical power to test variation in post‐secondary schooling.

Moving forward, these study findings should instigate significantly more attention to how early‐life course exposures and experiences may shape the incidence of AD and non‐AD dementia in later life. The main challenge for this research, however, is the limited existing data that include rich life‐course measures of both the early‐life risk factors and later‐life dementia outcomes. The study used here, the WLS, provides a unique opportunity to further explore not only additional early‐life exposures and experiences, but also how midlife experiences, ranging from occupational exposures to social relationships, may mediate or moderate those influences.

In conclusion, we find that early‐life risk factors that precede the completion of high school, rather than early adulthood schooling, exert the greatest influence on later‐life dementia risk, with differential mechanisms operating across dementia etiologies. Using education as a proxy for early‐life exposures conceals specific childhood mechanisms, like socioeconomic resources and both adolescent cognition and academic performance (independent from levels of schooling), that are separately intervenable. Moreover, because attainment also captures early to mid‐adulthood, it muddies our understanding of specific pathways shaping differential dementia risks. Our findings indicate that education's influence is confined to the early‐life period, where brain development is especially malleable.

## CONFLICT OF INTEREST STATEMENT

The authors report no conflicts of interest. All author disclosures are available in the Supporting Information.

## CONSENT STATEMENT

Participants provided informed consent for their participation in the Wisconsin Longitudinal Study.

## Supporting information



Supporting information

Supporting information
