# Supplementary material for: Early‐life influences on the risk for later‐life Alzheimer's and non‐Alzheimer's dementia: A nearly full life course prospective cohort study
Source: Alzheimers Dement. 2026 Jan 15;22(1):e70967. doi: 10.1002/alz.70967 (PMC12805529; doi:10.1002/alz.70967)
Supplement: Supplementary file 1 — Supporting information [file ALZ-22-e70967-s002.docx]

| Appendix Table A.1. Full Multinomial Model Estimates |  |  |  |
| --- | --- | --- | --- |
| [Reference Category: Those classified as having normal cognitive functioning] | | | |
|  |  |  |  |
| Consensus Diagnosis-Mild Cognitive Impairment | |  |  |
| Female | 0.59 [0.45,0.77] | 0.60 [0.46,0.79] | 0.64 [0.49,0.85] |
| Age at assessment (in 2021) | 1.12 [1.07,1.17] | 1.11 [1.06,1.17] | 1.11 [1.06,1.17] |
| APOE score | 1.40 [1.16,1.68] | 1.38 [1.15,1.67] | 1.39 [1.15,1.68] |
| Mother's Education | 0.98 [0.93,1.03] | 1.02 [0.97,1.07] | 1.02 [0.96,1.07] |
| Parental income '57 (ihs) | 0.93 [0.84,1.04] | 1.00 [0.89,1.11] | 0.99 [0.89,1.11] |
| Adolescent Cognition (std) |  | 0.56 [0.47,0.66] | 0.58 [0.47,0.71] |
| Years of education (std) |  |  | 1.06 [0.91,1.22] |
| High School Rank |  |  | 0.91 [0.76,1.08] |
| _cons | 0.11 [0.05,0.23] | 0.06 [0.03,0.12] | 0.06 [0.03,0.13] |
|  |  |  |  |
| Consensus Diagnosis-Non-AD Dementia | |  |  |
| Female | 0.58 [0.33,0.99] | 0.58 [0.34,1.00] | 0.53 [0.29,0.99] |
| Age at assessment (in 2021) | 1.20 [1.10,1.30] | 1.19 [1.10,1.28] | 1.18 [1.10,1.28] |
| APOE score | 1.29 [0.88,1.91] | 1.30 [0.88,1.90] | 1.29 [0.87,1.89] |
| Mother's Education | 0.92 [0.84,1.01] | 0.92 [0.83,1.01] | 0.92 [0.84,1.02] |
| Parental income '57 (ihs) | 0.78 [0.63,0.98] | 0.78 [0.62,0.98] | 0.79 [0.63,1.00] |
| Adolescent Cognition (std) |  | 1.09 [0.82,1.45] | 1.14 [0.82,1.59] |
| Years of education (std) |  |  | 0.81 [0.61,1.09] |
| High School Rank |  |  | 1.07 [0.76,1.50] |
| _cons | 0.06 [0.02,0.22] | 0.07 [0.02,0.23] | 0.06 [0.02,0.21] |
|  |  |  |  |
| Consensus Diagnosis-AD Dementia |  |  |  |
| Female | 1.18 [0.88,1.57] | 1.19 [0.89,1.59] | 1.46 [1.07,2.00] |
| Age at assessment (in 2021) | 1.22 [1.16,1.28] | 1.22 [1.16,1.28] | 1.22 [1.16,1.28] |
| APOE score | 2.09 [1.70,2.56] | 2.08 [1.68,2.57] | 2.11 [1.71,2.60] |
| Mother's Education | 1.03 [0.97,1.10] | 1.06 [0.99,1.13] | 1.05 [0.99,1.12] |
| Parental income '57 (ihs) | 0.90 [0.80,1.00] | 0.94 [0.84,1.06] | 0.93 [0.82,1.04] |
| Adolescent Cognition (std) |  | 0.65 [0.55,0.77] | 0.76 [0.62,0.93] |
| Years of education (std) |  |  | 1.17 [0.98,1.39] |
| High School Rank |  |  | 0.71 [0.58,0.86] |
| _cons | 0.02 [0.01,0.04] | 0.01 [0.00,0.03] | 0.01 [0.00,0.02] |
|  |  |  |  |
| r2_p | 0.07 | 0.09 | 0.09 |
| N | 4514.00 | 4514.00 | 4514.00 |
|  |  |  |  |
| Notes: |  |  |  |
| Standard errors in parentheses. |  |  |  |
| Dependent variable is dementia diagnosis in 2021. | |  |  |
| For those assessed based on the Dementia Questionnaire proxy instrument, age is calculated based on the age of the participant for | | | |
| those still alive, and age at death for those deceased. | |  |  |
| MCI findings are not included in the main table because the TICS-m is not sufficiently sensitive to fully capture those at risk for MCI | | | |
